# Valproic acid attenuates cellular senescence in diabetic kidney disease through the inhibition of complement C5a receptors

**DOI:** 10.1038/s41598-022-24851-w

**Published:** 2022-11-24

**Authors:** Melinda T. Coughlan, Mark Ziemann, Adrienne Laskowski, Trent M. Woodruff, Sih Min Tan

**Affiliations:** 1 Department of Diabetes, Central Clinical School, Monash University, Melbourne, VIC Australia; 2 School of Life and Environmental Sciences, Deakin University, Geelong, VIC Australia; 3 Drug Discovery Biology, Monash Institute of Pharmaceutical Science, Monash University Parkville Campus, Melbourne, VIC Australia; 4 School of Biomedical Science, Faculty of Medicine, The University of Queensland, St Lucia, Brisbane, Australia

**Keywords:** Physiology, Nephrology, Pathogenesis

## Abstract

Despite increasing knowledge about the factors involved in the progression of diabetic complications, diabetic kidney disease (DKD) continues to be a major health burden. Current therapies only slow but do not prevent the progression of DKD. Thus, there is an urgent need to develop novel therapy to halt the progression of DKD and improve disease prognosis. In our preclinical study where we administered a histone deacetylase (HDAC) inhibitor, valproic acid, to streptozotocin-induced diabetic mice, albuminuria and glomerulosclerosis were attenuated. Furthermore, we discovered that valproic acid attenuated diabetes-induced upregulation of complement C5a receptors, with a concomitant reduction in markers of cellular senescence and senescence-associated secretory phenotype. Interestingly, further examination of mice lacking the C5a receptor 1 (C5aR1) gene revealed that cellular senescence was attenuated in diabetes. Similar results were observed in diabetic mice treated with a C5aR1 inhibitor, PMX53. RNA-sequencing analyses showed that PMX53 significantly regulated genes associated with cell cycle pathways leading to cellular senescence. Collectively, these results for the first time demonstrated that complement C5a mediates cellular senescence in diabetic kidney disease. Cellular senescence has been implicated in the pathogenesis of diabetic kidney disease, thus therapies to inhibit cellular senescence such as complement inhibitors present as a novel therapeutic option to treat diabetic kidney disease.

## Introduction

The International Diabetes Federation estimated that 537 million adults, or 1 in 10 people, are living with diabetes in 2021^1^. Chronic kidney disease associated with diabetes, also known as diabetic kidney disease (DKD), is the leading cause of end stage renal disease (ESRD) in many developed countries^2^. It is estimated that 30% of patients with type 1 diabetes mellitus and 40% of patients with type 2 diabetes mellitus will develop DKD^3^. Current treatments for DKD focuses on anti-hypertensive therapies including angiotensin-converting enzyme (ACE) inhibitors and angiotensin receptors blockers (ARBs), strict glycemic control with anti-diabetic agents such a metformin and more recently, sodium-glucose cotransporter 2 (SGLT2) inhibitors^4,5^. Nonetheless, the majority of these therapies delay, but do not reduce the risk of progression to DKD^3^. Therefore, there remains a critical need to identify new therapeutic targets to treat and prevent the development of DKD.

In the search for novel therapies for diabetic kidney disease, there is also interest in drug repurposing opportunities, including the study of histone deacetylase (HDAC) inhibitors^6^. One of the HDAC inhibitors, valproic acid has shown promising renoprotective effects in preclinical studies of diabetic kidney disease^7,8^. Valproic acid has been shown to induce histone acetylation and modulate DNA and histone methylation status, thus altering the expression of transcription factors leading to regulation of gene expression^9^.More recently, an in vitro study has shown that valproic acid can attenuate hyperglycemia-induced activation of complement cascade genes in HepG2 human hepatocytes^10^. However, whether valproic acid attenuates complement cascade genes in an in vivo setting is unknown. This study aims to investigate if valproic acid mediates its renoprotective effects via the inhibition of the complement cascade in streptozotocin-induced diabetic mice.

Complement is an integral part of the innate immune system. Our group has recently identified complement C5a acting via its receptor, C5aR1, is a key pathogenic driver of DKD^11^. Importantly, this pathway is not targeted by conventional therapy for DKD, as individuals with diabetes receiving ARBs still exhibited high levels of the circulating complement endproduct, C5a^11^. We have recently reported that C5a-C5aR1 signaling induces changes in mitochondrial agility in DKD, specifically through changes in cardiolipin remodeling, mitochondrial metabolite flux, and mitochondrial respiratory function^11^. While the precise mechanisms of how C5a and its receptors mediate renal injury in diabetes are only starting to be unraveled^12^, early evidence suggests that it is an attractive therapeutic target^11,13,14^.

Recently, a study demonstrated that C5a, induces tubular senescence and the development of senescence associated secretory phenotype (SASP) in renal tubular epithelial cells (RTEC) after renal ischaemia reperfusion injury^15^. This is partly due to aberrant DNA methylation induced by C5a in the RTEC, particularly in regions involved in cell cycle control and DNA damage^15^. There is increasing evidence to support a pathogenic role for senescent renal cells in inducing SASP in DKD^16^. However, whether C5a is involved in inducing cellular senescence and SASP in DKD is unclear. Thus, we sought to investigate whether C5a plays a role in inducing cellular senescence in our preclinical models of DKD.

## Results

### Valproic acid improves glycemic control in type 1 diabetes

**Figure 1:**
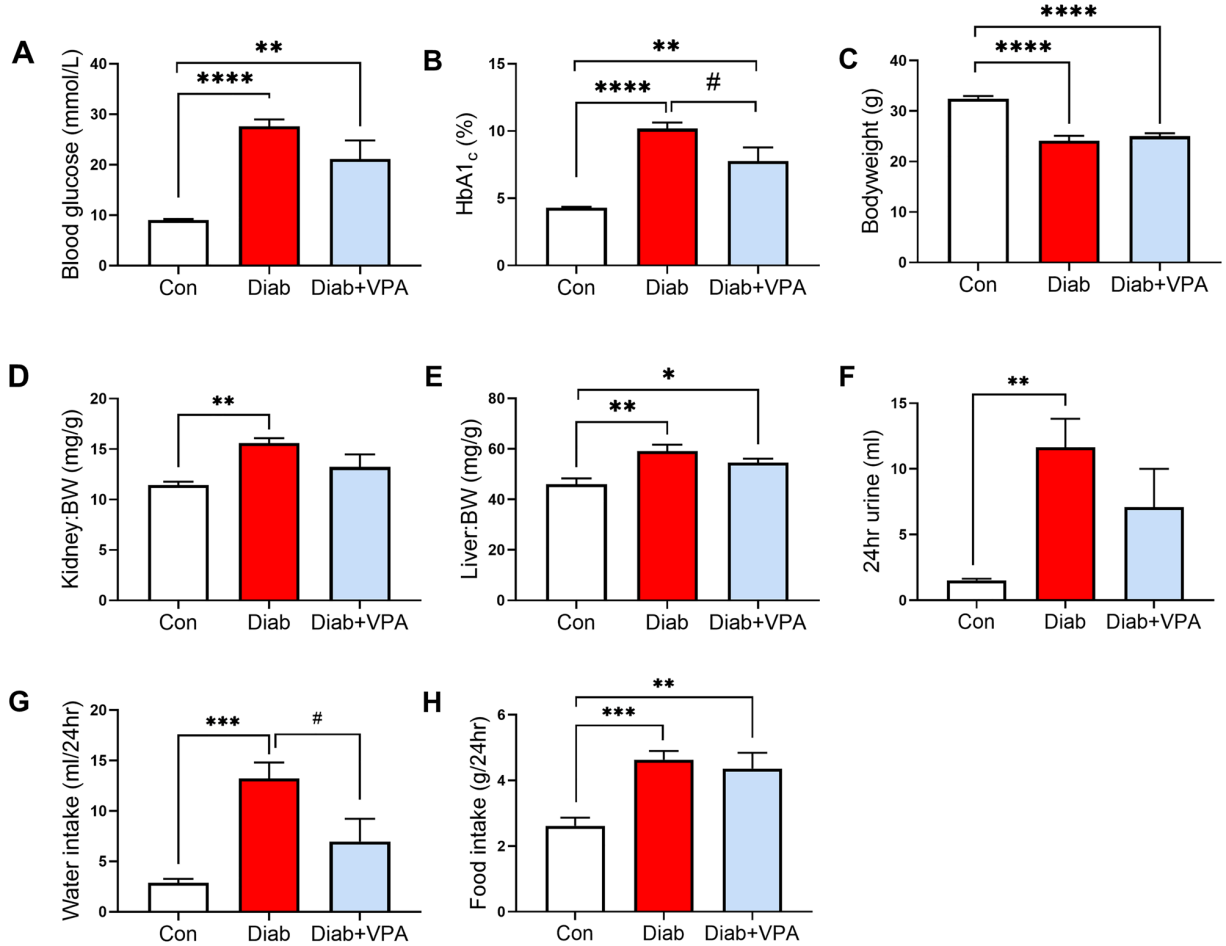
VPA improves glycemic control in STZ mice. (**A**) Blood glucose, (**B**) HbA1c, (**C**) body weight, (**D**) kidney to body weight ratio, (**E**) liver weight to body weight ratio, (**F**) urine output, (**G**) water intake and (**H**) food intake were measured in diabetic mice treated with VPA. *P < 0.05, **P < 0.01, ***P < 0.001, ****P < 0.0001 vs Con; ^#^P < 0.05 vs Diab. Data are presented as mean ± SEM, n = 7–9 mice per group.

As expected, streptozotocin-induced diabetic mice displayed a significant increase in blood glucose (Fig. 1A) and HbA1c (Fig. 1B). Importantly, valproic acid significantly reduced HbA1c in diabetic mice suggesting an improvement in glycemic control (Fig. 1B). There was a significant decrease in body weight in streptozotocin-induced diabetic mice (Fig. 1C) and a significant increase in kidney to body weight ratio (Fig. 1D) suggesting renal hypertrophy, however these parameters were not affected by valproic acid treatment. Liver weight was increased in diabetes irrespective of treatment (Fig. 1E). There was a reduction in 24-h urine output (Fig. 1F) and water intake (Fig. 1G) in valproic acid-treated diabetic mice when compared to vehicle, which coincided with diabetes status. Valproic acid treatment did not affect food intake in the diabetic mice (Fig. 1H).

### Valproic acid attenuates diabetes-induced albuminuria and renal injury

**Figure 2:**
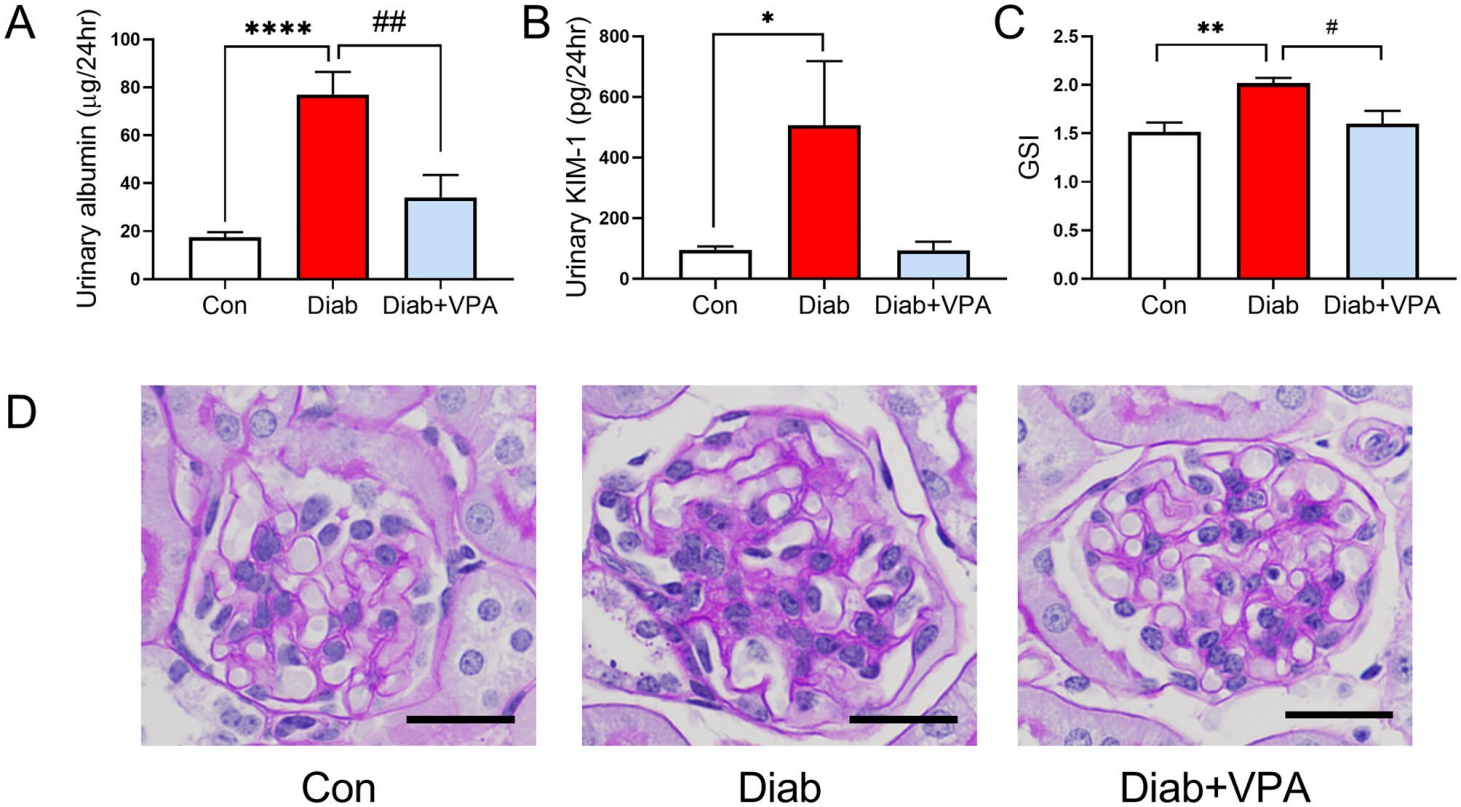
VPA treatment attenuates renal injury in diabetes. Albuminuria (**A**), tubular injury marker, KIM-1 (**B**) and glomerulosclerosis marker, GSI (**C**) were attenuated by 8 weeks of VPA treatment. (**D**) Representative micrographs of PAS-stained glomeruli. Original magnification × 400, scale bar = 25 µm. *P < 0.05, **P < 0.01, ****P < 0.0001 vs Con; ^#^P < 0.05, ^##^P < 0.05 vs Diab. Data are presented as mean ± SEM, n = 7–9 mice per group.

Valproic acid has previously been shown to be renoprotective in streptozotocin-induced diabetic rats^7,17^. In the current study, 10 weeks after streptozotocin treatment, there was a significant increase in albuminuria (Fig. 2A), tubular injury marker, KIM-1 (Fig. 2B), and glomerulosclerosis (Fig. 2C,D). Treatment of diabetic mice with valproic acid for 8 weeks significantly attenuated these renal injury parameters (Fig. 2).

### Valproic acid reduces complement C5a receptors in streptozotocin-induced type 1 diabetes

**Figure 3:**
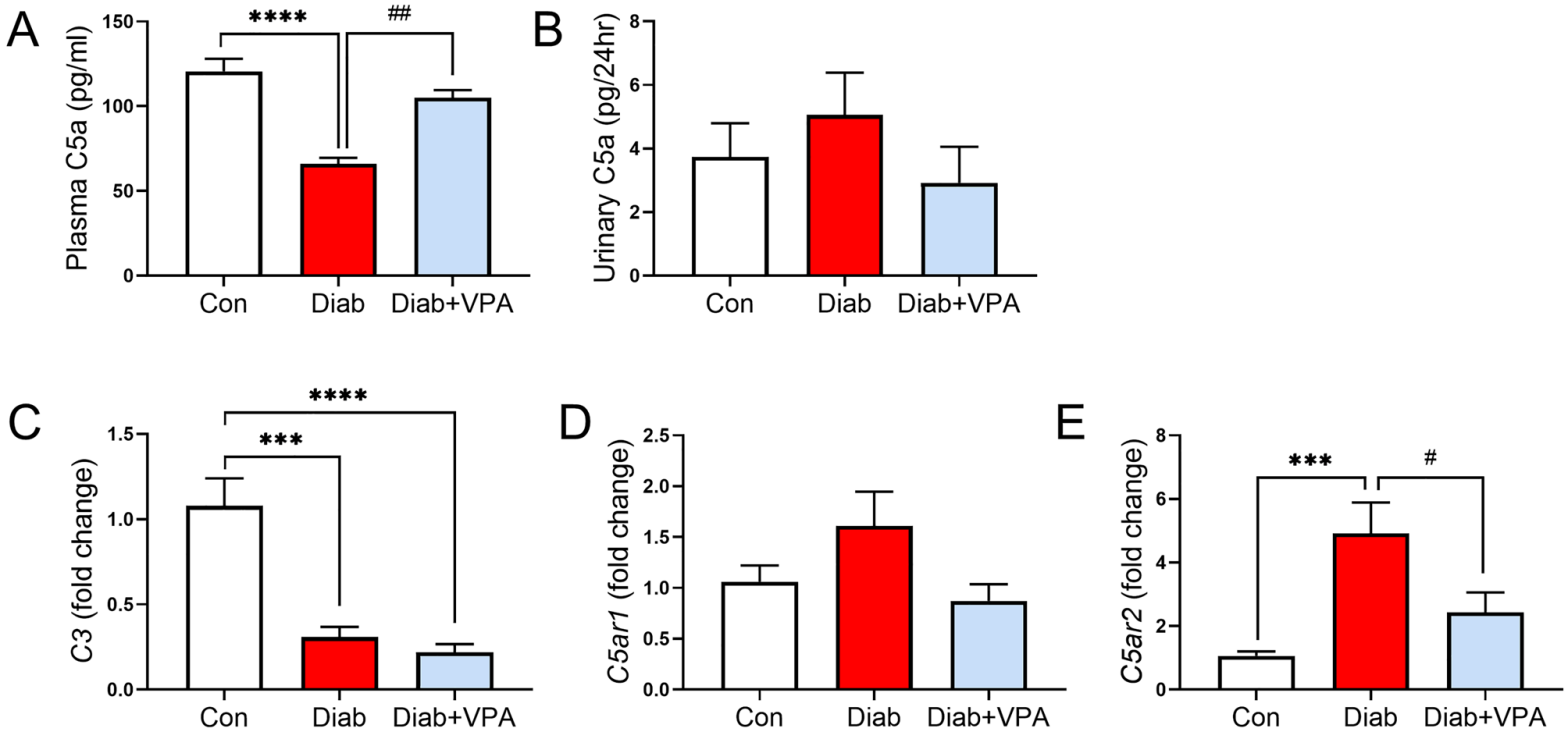
Effects of VPA treatment on Complement in diabetes. (**A**) Plasma and (**B**) urine C5a were measured using ELISA. Renal cortical *C3* (**C**), *C5ar1* (**D**) and *C5ar2* (**E**) were measured by RT-qPCR. ***P < 0.001, ****P < 0.0001 vs Con; ^#^P < 0.05, ^##^P < 0.051vs Diab. Data are presented as mean ± SEM, n = 7–9 mice per group.

There was a reduction in circulating C5a in diabetes and this was restored by valproic acid (Fig. 3A), while no significant changes were observed in urinary C5a levels across all treatment groups (Fig. 3B). Unlike our previous long term diabetes study which showed an upregulation of *C3* expression in the renal cortex at 24 weeks of diabetes duration^11^, *C3* was significantly downregulated 10 weeks after streptozotocin treatment (Fig. 3C). Valproic acid treatment did not result in any changes in *C3* expression in the diabetic kidney. However, valproic acid inhibited the diabetes-induced upregulation of C5a receptor gene expression (Fig. 3D,E) in the renal cortex, although the reduction in *C5ar1* did not reach statistical significance (P = 0.0822). Importantly, a reduction in circulating C5a but an increased in C5a receptors in the kidneys suggest a local kidney activation of this terminal pathway of the complement cascade at 10 weeks post diabetes induction.

### Valproic acid inhibits tubular senescence in early DKD

**Figure 4:**
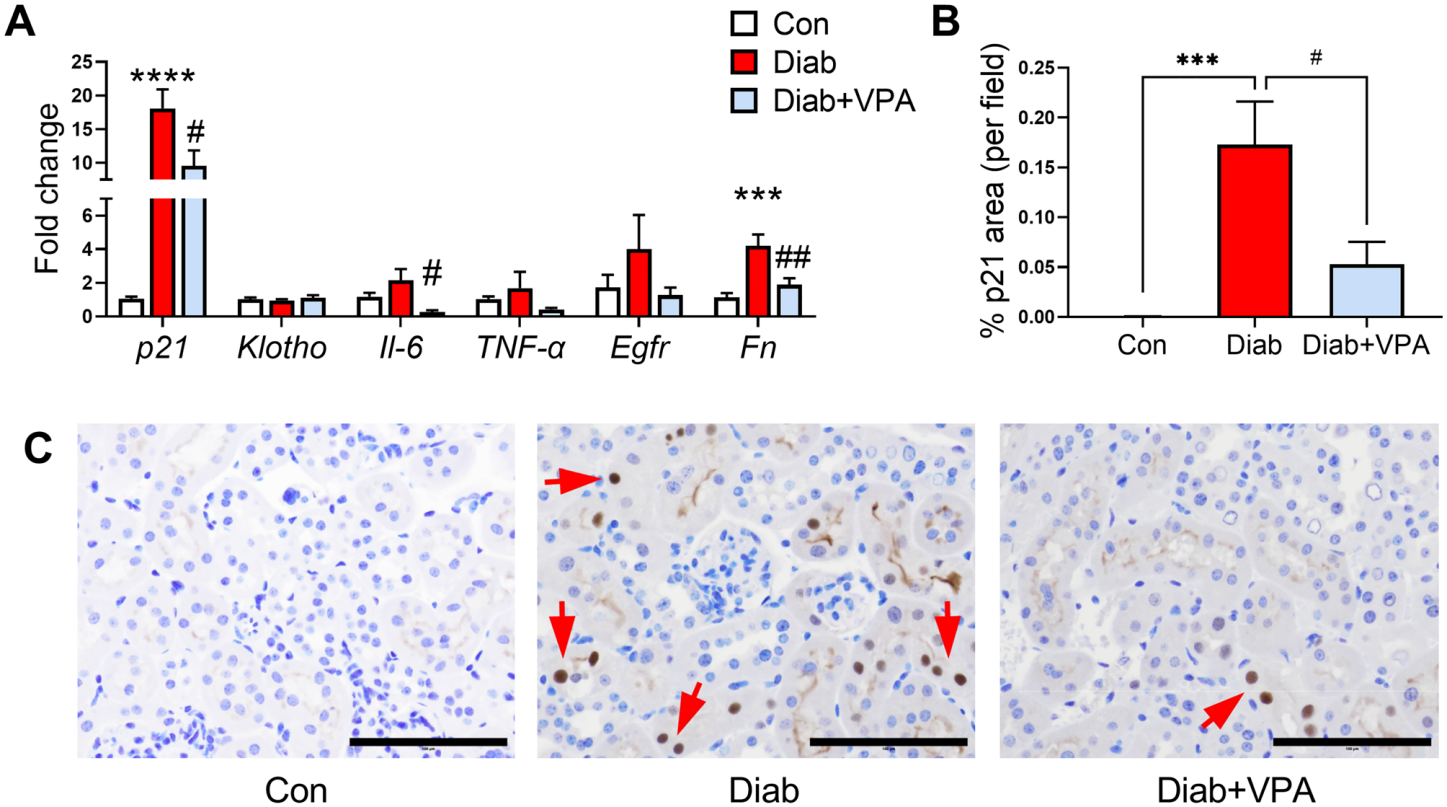
VPA attenuates diabetes-induced cellular senescence in the kidney cortex. (**A**) Senescence markers and SASP factors were measured by RT-qPCR. (**B**) p21 was measured by immunohistochemistry. (**C**) Representative micrographs of p21 immunostaining. Original magnification × 200, scale bar = 100 µm. ***P < 0.001, ****P < 0.0001 vs Con; ^#^P < 0.05, ^##^P < 0.01 vs Diab. Data are presented as mean ± SEM, n = 7–9 mice per group.

Interestingly, we found that valproic acid had an effect on diabetes-induced cellular senescence. p21^waf1/^cip1 or p21 is a cyclin-dependent kinase inhibitor that mediates p53-dependent cell cycle arrest in senescent cells^18^. Treatment of valproic acid for 8 weeks significantly attenuated diabetes-induced expression of *p21* (Fig. 4A) and the SASP markers *Il-6*, *TNF-α*, *EGFR* and *Fn* (Fig. 4A). However, *Klotho* expression was not altered in the mice after 10 weeks of diabetes (Fig. 4A). Furthermore, we found the levels of p21 to be significantly increased in tubular and interstitial cells in the renal cortex of diabetic mice when compared to nondiabetic controls (Fig. 4B) and this was attenuated by valproic acid (Fig. 4C). Collectively, these results indicate that valproic acid attenuated diabetes-induced cellular senescence.

### Inhibition of C5aR1 attenuates senescence and SASP in the kidney

**Figure 5:**
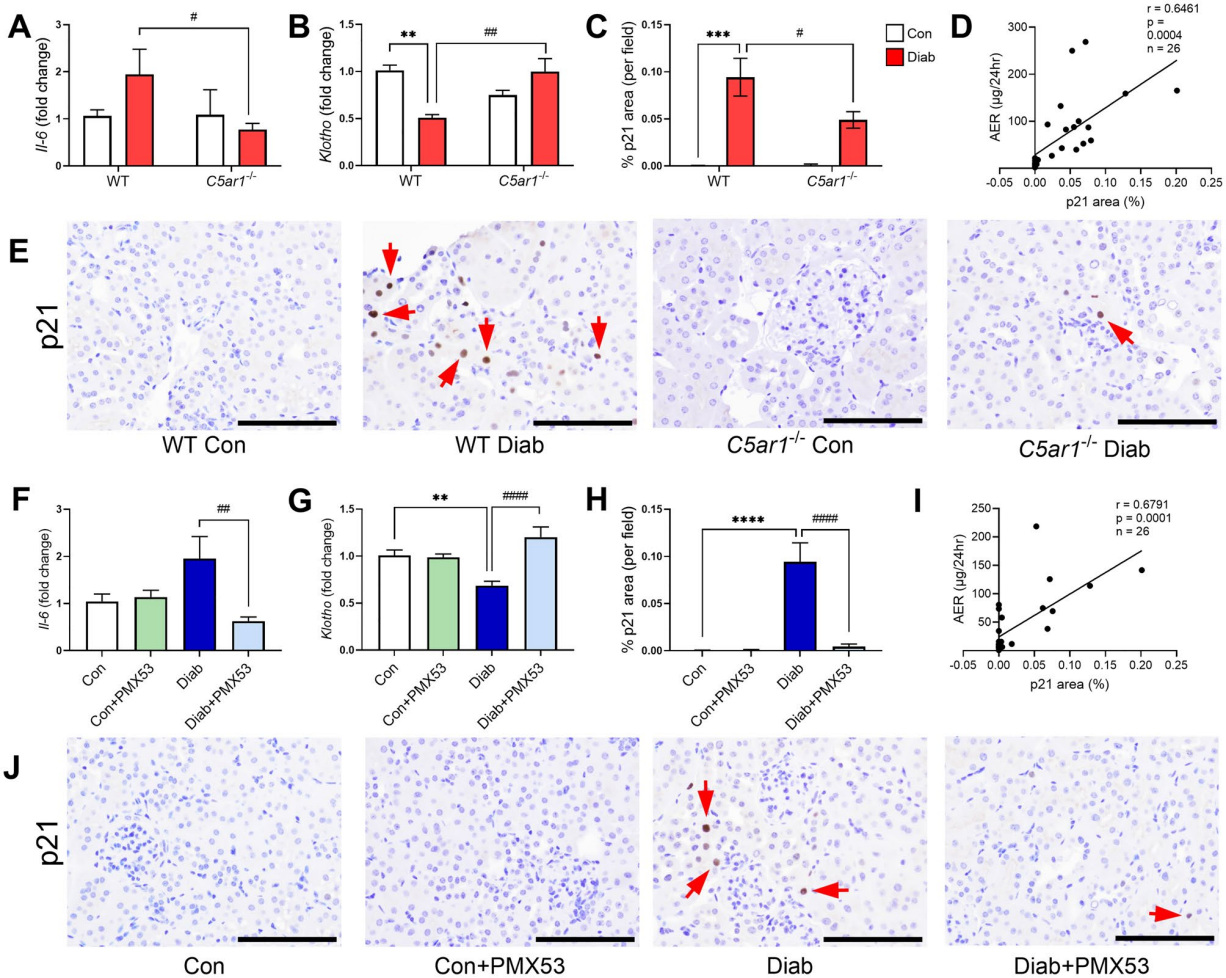
Inhibition of C5aR1 attenuates cellular senescence and SASP in DKD. In *C5ar1*^−/−^ mice, SASP cytokine *IL-6* (**A**) was reduced and diabetes-induced loss of *Klotho* was restored (**B**). Senescence marker, p21 immunostaining was increased in renal tubular and tubulointerstitial cells (arrows) in diabetes and attenuated in *C5ar1*^−/−^ mice in the kidney cortex (**C**,**E**). Levels of p21 in these mice were correlated with albuminuria (**D**). Similarly, inhibition of C5aR1 by PMX53 in diabetic mice attenuated *IL-6* (**F**) and loss of *Klotho* (**G**) in the kidney cortex. Immunostaining of p21 (**H**,**J**) was increased in diabetes and reduced by PMX53 and was significantly correlated with albuminuria (**I**). Original magnification × 200, scale bar = 100 µm. **P < 0.01, ***P < 0.001, ****P < 0.0001 vs Con; ^#^P < 0.05, ^##^P < 0.01, ^####^P < 0.0001 vs Diab. Data are presented as mean ± SEM, n = 4–12 mice per group.

In order to investigate if the effects of valproic acid on cellular senescence are via activation of complement receptors, we examined cellular senescence markers in a preclinical model of DKD where C5aR1 was either genetically deleted or inhibited with a pharmacological antagonist. We have previously shown that inhibition of C5aR1 with a pharmacological inhibitor, PMX53, or genetic deletion of the *C5ar1* gene attenuated the development of albuminuria, renal injury and inflammation in chemical-induced diabetes (streptozotocin-induced diabetic mice) after 24 weeks of diabetes^11^. PMX53 is a highly specific, orally active cyclic peptide antagonist of C5aR1 and does not bind to or inhibit the other C5a receptor, C5aR2^19^. Here, we studied whether inhibition of C5aR1 affected cellular senescence in DKD. SASP marker, IL-6, was increased in renal cortex of mice with diabetes (Fig. 5A,F) and its gene expression was significantly decreased by the genetic deletion of C5aR1, in C5aR1 knockout (KO) mice (Fig. 5A,F). We found that renal cortical *Klotho* expression is significantly downregulated in diabetes and its expression was restored by the deletion of C5aR1 (Fig. 5B,G). Protein expression of p21 in the renal cortex was attenuated by PMX53 (Fig. 5C,E), and, in the absence of *C5ar1* expression (Fig. 5H,J). Collectively, these results suggest a role for C5aR1 in mediating tubular senescence in DKD. Importantly, the levels of p21 were positively correlated with albuminuria in these mice (Fig. 5D,I), further supporting the notion that diabetes-induced senescence may play a role in mediating renal injury in DKD.

### Transcriptomic pathway analysis reveals changes in cell cycle and senescence pathways

**Figure 6:**
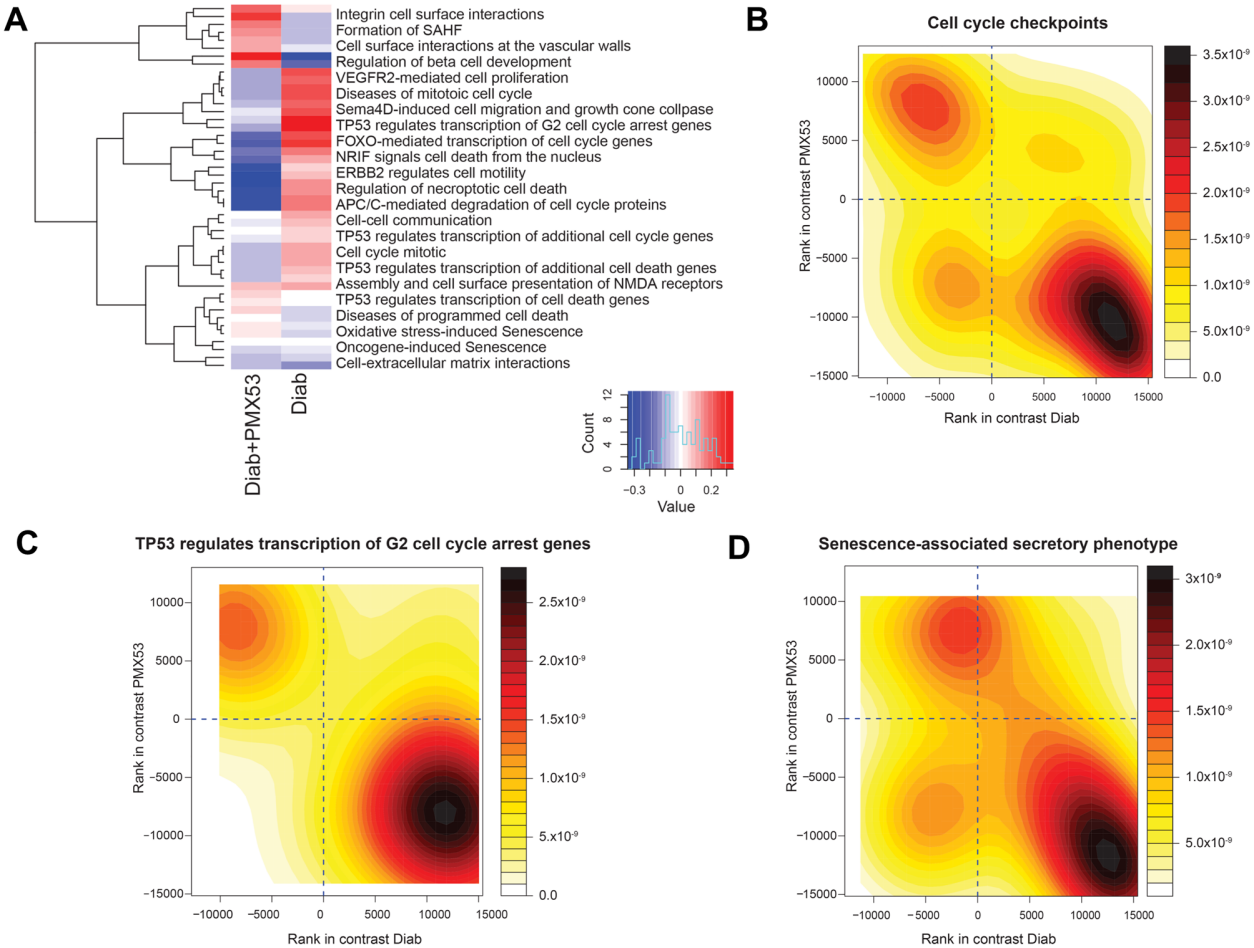
RNA sequencing analysis reveal a role for C5aR1 in modulating cellular senescence in the diabetic kidney. (**A**) Heatmap of the most significant differentially expressed genes in cell cycle and senescence pathways. Rank-rank plots of gene expression changes in (**B**) cell cycle checkpoints, (**C**) TP53 regulates transcription of G2 cell cycle arret genes and (**D**) senescence-associated secretory phenotype. Both heatmap and rank-rank plots are produced by Mitch in R (https://bioconductor.org/packages/mitch/).

We performed whole kidney cortex transcriptomic analysis using RNA-sequencing in diabetic mice treated with PMX53 after 24 weeks of diabetes. Reactome gene set analysis identified that cell cycle and senescence networks were modified by PMX53 treatment (Fig. 6A). Specifically, pathways associated with TP53-regulated transcription of cell cycle genes were upregulated in diabetes and downregulated by PMX53 (Fig. 6A). TP53 encodes for p53 which is a transcription factor integral in regulating genes involved in metabolism, autophagy, DNA damage repair, cell cycle arrest, senescence, and apoptosis^20^. One of the most important roles for p53 is the induction of cell cycle arrest following DNA damage through p21, leading to cellular senescence^21^. Rank-rank density plot analysis also showed differential genes associated with cell cycle checkpoint (Fig. 6B), TP53 regulates transcription of G2 cell cycle arrest genes (Fig. 6C) and senescence-associated secretory phenotype (Fig. 6D). These analyses revealed that a significant proportion of genes associated with these pathways were upregulated in diabetes and downregulated by PMX53, further supporting the involvement of C5aR1 in mediating diabetes-induced cell cycle arrest and senescence.

## Discussion

In this study, we have shown that valproic acid is renoprotective in a short-term mouse model of streptozotocin-induced diabetes. Furthermore, valproic acid attenuated diabetes-induced upregulation of *C5ar1* and *C5ar2* expression, concomitant with a downregulation of cellular senescence markers. To investigate if C5a is involved in mediating cellular senescence, we looked at cellular senescence markers in our previous long term diabetes models and we found that inhibition of C5aR1 attenuated diabetes-induced cellular senescence in DKD. These studies thus show for the first time that C5a plays a role in mediating cellular senescence in DKD and further validate complement as a novel therapeutic target for DKD.

Valproic acid is the most frequently prescribed antiepileptic drug and has been successfully used for other indications including bipolar disorder, migraine headache, and diabetic neuropathy-related pain. Although first prescribed almost 60 years ago as an epilepsy treatment, its mechanism of action is still unclear. The most commonly proposed mechanisms are by acting on γ aminobutyric acid (GABA) levels in the central nervous system, blocking voltage-gated ion channels, and inhibiting HDAC^22^. It is through its inhibitory action on HDAC that valproic acid was discovered to be antidiabetic^23,24^ and renoprotective in DKD^7,8^. Valproic acid inhibits HDACs categorized as class Ia (HDAC1 and HDAC2), class Ib (HDAC3), class Ic (HDAC8), and class IIa (HDAC4, HDAC5, and HDAC7), leading to an increase in the acetylation of histones H2, H3, and H4, which modulates the expression of associated genes^25^. More recently, we have shown valproic acid was able to attenuate hyperglycemia-induced activation of complement genes in hepatocytes^10^. In this study, we found that valproic acid also attenuated diabetes-induced upregulation of complement genes, such as C5aR1 and C5aR2, in the diabetic kidney, suggesting a new pathway regulated by valproic acid. Interestingly, in this short-term diabetes study, the majority of complement genes were downregulated as opposed to what we observed in a longer-term diabetes study of 24 weeks after STZ^11^. Nonetheless, expression of the receptor *C5ar2* was upregulated. Furthermore, valproic acid treatment attenuated diabetes-induced upregulation of *C5ar2*. While we have previously demonstrated a pathological role for C5aR1 in DKD, the role of C5aR2 is currently unknown. Thus, whether the inhibition of C5aR2 by valproic acid contributes to a functional phenotype requires further investigation.

Valproic acid is thought to exhibit potent antitumor properties by modulating multiple pathways including cell cycle arrest, apoptosis and senescence^26^. In our study, we have shown that valproic acid downregulates markers of cellular senescence and SASP. However, these effects are likely cell type specific and may depend on the level of differentiation and the underlying genetic alterations^26^. Indeed, valproic acid has been shown to exhibit both anti-senescence^27^ and pro-senescence properties^28,29^. Nonetheless, we have shown that valproic acid exerts its renoprotective effects in our short-term diabetes study possibly by modulating complement genes and attenuating cellular senescence.

Diabetes is associated with senescence in a “malignant” positive feedback where metabolic dysfunction in prediabetes leads to cellular senescence and the accumulation of senescent cells contributes to further metabolic dysfunction, inflammation and tissue injury^30^. Accumulating evidence suggests that premature aging of the kidney occurs in DKD. Verzola and colleagues demonstrated that biopsies from patients with DKD showed marked increase of senescent cell markers, p16^INK4A^ and SA-β-Gal, mostly in the tubule cells and to a lesser extent, podocytes compared with age-matched controls, indicating that an age effect could not be responsible for the findings^31^. Furthermore, accelerated senescence was already observed in proteinuric patients with normal/subnormal eGFR, suggesting that cellular senescence is an early occurrence in disease progression. In our study, senescent marker, p21, is increased in the kidney cortex 10 weeks after diabetes induction, where mice only exhibit mild albuminuria and glomerulosclerosis. Furthermore, previous animal studies have shown the appearance of senescent cells as early as 10 days in rats and 4 weeks in C57BL/6J mice after streptozotocin treatment^32,33^. Collectively, these findings suggest that intervention targeting senescent cells prior to the development of DKD might be preventative.

A state of chronic, sterile, low grade inflammation, termed inflammaging has been associated with chronic metabolic diseases, such as type 2 diabetes (T2D)^34^. It is thought that cellular senescence and SASP are central players in inflammaging in diabetes and its complications^35^. The link between inflammaging and the complement system has recently been explored in the context of acute kidney injury and chronic graft damage^36^. We and others have previously demonstrated a link between complement-induced inflammation and the pathogenesis and progression of DKD^37,38^. However, the role of the complement system in inflammaging and senescence in DKD is less well known. Previous studies have reported a role for C5a in cellular senescence^15,39^. Castellano and colleagues first reported that C5a induced cellular senescence through epigenetic modifications in renal tubular epithelial cells (RTEC)^15^. Hypomethylation of regions involved in aging pathways such as Wnt/βcatenin led to an upregulation of senescence marker SA-β Gal and cell cycle arrest markers, p53 and p21^15^. In this study, we showed for the first time that complement C5a is able to induce cellular senescence in DKD. Inhibition of C5aR1 led to an attenuation of senescence markers p21 and SASP cytokine IL-6 in the kidney cortex of diabetic mice. Furthermore, using RNA-Seq transcriptomic analyses, we demonstrated that inhibition of C5aR1 in diabetes rewired the cellular senescence network dampening signals associated with cell cycle checkpoints, TP53-regulated cell cycle and cell death pathway and SASP. In addition, there was a clear positive correlation between p21 in the kidney cortex and albuminuria, suggesting a functional role for C5a-induced senescence in DKD.

Klotho is an anti-aging gene that is expressed primarily in the distal collecting duct and proximal tubules of the kidney^40^. The decline and/or loss of klotho has been associated with human chronic kidney disease, with the levels of klotho positively correlated with eGFR^41^. Furthermore, decreased plasma klotho is a predictive marker for the progression of type 2 diabetic kidney disease^42^. Klotho is a pleiotropic protein that exhibits multifaceted functions related to kidney diseases. Most notably, it inhibits cellular senescence by suppressing Wnt signaling, which is a prominent inducer of cellular senescence^43^. Although research on the functions of klotho in chronic kidney diseases have gained considerable ground in the last decades, much is still unknown of the regulation of its expression, release, and metabolism. More recently, complement, specifically C5a, is thought to regulate klotho in renal IRI^44^. Stimulation of renal proximal tubular epithelial cells with C5a induced a marked reduction in *Klotho* through an NFκB-dependent mechanism^44^. In our study, loss of *Klotho* in the diabetic setting was restored in mice lacking C5aR1 expression or function in our long-term diabetes model of 24 weeks. This restoration of *Klotho* expression was associated with an inhibition of cellular senescence and improvement of kidney function in diabetic mice, suggesting a role of complement in the regulation of klotho in chronic kidney disease, similar to what is observed in acute kidney injury. However, it should be noted that *Klotho* expression was not changed in our short-term diabetes model of 10 weeks, suggesting that the loss of Klotho occurs later in DKD disease progression.

Limitations of the present study include not including a valproic acid-treated non-diabetic control group. However, our previous study has shown that valproic acid does not cause adverse effects in hepatocytes cultured in normal glucose and valproic acid did not alter the expression of complement genes in the control group^10^. Furthermore, previous studies have shown no adverse renal effects of valproic acid in nondiabetic rats and mice^8,17^.

In conclusion, we have shown for the first time that complement C5a modulates cellular senescence in DKD via its receptor C5aR1. Although the precise mechanisms are yet to be determined, C5a regulation of the anti-senescence gene, *Klotho* appear to be of significance. This area of research is of clinical significance since the current preferred treatments for DKD including renin-angiotensin system inhibitors and SGLT2 inhibitors, have been shown not to target the complement cascade^11^ or cellular senescence^45^. We have shown that the HDAC inhibitor, valproic acid, modulates complement cascade genes which partly contributes to its renoprotective effects in DKD. However, mechanisms of valproic acid is complex and is associated with many side effects including gastrointestinal discomfort, weight gain, tremor, hair loss, thrombocytopenia, infertility, and teratogenicity^24^. Although these side effects can be managed on a patient-to-patient basis, therapy to directly inhibit complement activation may present as a more attractive option. Importantly, C5aR1 inhibitors such as PMX53 have been proven safe and well-tolerated in Phase I clinical trials^24^. Increasing evidence indicates a pivotal role for complement in the pathogenesis of DKD. With the more recent discovery of the epigenetic role for C5a in cellular senescence and its role in mediating kidney injury, inhibition of C5a has once again proven to be an important therapeutic target for DKD.

## Methods

### Animal experiments

All animal experiments were approved by the Alfred Research Alliance Animal Ethics Committee (Ethics approval numbers: E/0926/2010/B and E/1870/2018/M) and performed in accordance with guidelines from the National Health and Medical Research Council of Australia. All rodents were housed in a temperature-controlled environment, with a 12-h light/12-h dark cycle, and had access to chow (Specialty Feeds, Perth, Western Australia, Australia) and water ad libitum. C57BL/6J and *C5ar1*^−/−^ mice backcrossed onto a C57BL/6J background (gift from Professor Rick Wetsel, University of Texas) were bred at Alfred Research Alliance Animal Services. Diabetes was induced in 6-week-old mice by five daily intraperitoneal injections of low-dose streptozotocin (STZ; 55 mg/kg) (Sigma-Aldrich, St. Louis, MO). Mice in the nondiabetic group were given 0.5 M sodium citrate. For the knockout study, diabetic and nondiabetic WT mice (n = 7–10) and *C5ar1*^−/−^ mice (n = 4–12) were followed for 24 weeks after the onset of diabetes. For the PMX53 study, diabetic and nondiabetic mice were randomized to receive either 1) the C5aR1 peptide inhibitor PMX53 (Ac-Phe-[Orn-ProdCha-Trp-Arg]); synthesized as previously described^46^ at 2 mg/kg body weight, administered in the drinking water, or 2) drinking water alone (n = 6–12 mice). These mice were followed for 24 weeks. For the VPA study, diabetic mice were randomized to receive VPA at 150 mg/kg body weight daily via oral gavage (n = 7) two weeks after STZ treatment, and the vehicle group was given water (n = 8). Nondiabetic controls were given water via oral gavage daily (n = 9). These mice were followed for 8 weeks. At the end of the study, plasma and kidneys were collected for analysis. The study is reported in accordance with ARRIVE guidelines.

### Assessment of renal function and metabolic parameters

Mice were housed singly in metabolic cages a week before the end of the study to collect 24-h urine and food and water intake recorded. Renal function and metabolic parameters for *C5ar1*^−/−^ and PMX53 studies have been previously published^11^. For the VPA study, plasma glucose was measured using a glucose colorimetric assay kit (Cayman Chemical). Glycated hemoglobin was measured using a Cobas Integra 400 Autoanalyzer (Roche Diagnostics Corp.). Urinary albumin was measured using a mouse albumin ELISA kit (Bethyl Laboratories, Montgomery, TX). Urinary and plasma C5a was measured using a mouse Complement Component C5a ELISA kit (RayBiotech) and urinary KIM-1 was measured using a mouse KIM-1 ELISA kit (Cloud-clone Corp).

### Renal histology

Paraffin-embedded kidney sections (3 μm thick) were stained with periodic acid Schiff (PAS) and scored for glomerulosclerotic index (GSI) as previously described^11^.

### Immunohistochemistry

Paraffin sections of mouse kidney (4 mm thick) were immunostained for p21 (Abcam). Briefly, endogenous peroxidases were blocked with 3% hydrogen peroxide for 15 min and incubated in 0.5% skim milk/Tris-buffered saline for 1 h at room temperature. The primary antibody was left on overnight at 4 °C. This was followed by incubation with biotinylated secondary antibody at room temperature for 10 min. Sections were then incubated with Vectastain ABC reagent (Vector Laboratories). Peroxidase activity was identified by reaction with 3,39-diaminobenzidine tetrahydrochloride (Sigma-Aldrich Pty. Ltd.). Sections were counterstained with hematoxylin. All sections were examined under an Olympus BX-50 light microscope (Olympus Optical) and digitized with a high-resolution camera. All digital quantitation (Image-Pro Plus software version 6.0) and assessments were performed in a blinded manner.

### Quantitative real-time RT-PCR

RNA from renal cortex was extracted using TRIzol Reagent, and cDNA was synthesized as described previously^47^. Gene expression was determined using a 7500 Fast Real-Time PCR System (Applied Biosystems, Victoria, Australia). Gene expression was normalized relative to 18S rRNA, and the relative fold difference in expression was calculated using the comparative 2^-ΔΔCt^ method.

### RNA sequencing and analysis

Previously described kidney RNA-Seq data from our laboratory (Tan et al.^11^), was downloaded from the NCBI GEO database under accession number GSE118089 (September 2021). Two contrasts were examined (i) control vehicle-treated compared to diabetic vehicle-treated and (ii) diabetic vehicle-treated compared to diabetic PMX53-treated. All sample groups contained n = 6 replicates. Genes with fewer than 10 reads per gene were omitted from downstream analysis. Differential expression analysis was conducted with DESeq2 (v1.32.0)^48^. Integrative pathway analysis was conducted using the Mitch package (v1.4.1; https://bioconductor.org/packages/mitch/)^49^ with default settings and gene sets downloaded from REACTOME on the 22nd September^50^. To focus on senescence-related pathways, we selected gene sets relevant to cell cycle, senescence and performed mitch once again.

### Statistical analysis

The data are expressed as mean ± SEM. Statistical analyses were performed using GraphPad Prism software version 8.0 (GraphPad Software). All data were analyzed with one-way ANOVA with the Tukey post hoc test, unless otherwise stated. A P value < 0.05 was considered statistically significant. All figure panels were made in GraphPad Prisms software version 8.0.

## Data Availability

Sequence data have been deposited in the National Center for Biotechnology Information Gene Expression Omnibus with accession number GSE118089.
